# Individualized conservative therapeutic strategies for adenomyosis with the aim of preserving fertility

**DOI:** 10.3389/fmed.2023.1133042

**Published:** 2023-03-30

**Authors:** Lei Han, Yanni Liu, Kaixue Lao, Jianxi Jiang, Caiying Zhang, Yanlin Wang

**Affiliations:** ^1^Department of Reproductive Medicine, Binzhou Medical University Hospital, Binzhou, Shandong, China; ^2^Department of Reproductive Medicine, Maternal and Child Health Hospital Affiliated to Zunyi Medical University, Zunyi, Binzhou, China; ^3^Department of Obstetrics and Gynecology, Binzhou Medical University Hospital, Binzhou, Shandong, China; ^4^Department of Postgraduate Student Office, Binzhou Medical University Hospital, Binzhou, Shandong, China

**Keywords:** adenomyosis, individualized treatment, uterus sparing operation, levonorgestrel-releasing intrauterine system, gonadotrophin releasing hormone agonist

## Abstract

Adenomyosis is a diffuse or localized organic disease caused by benign invasion of endometrial glands and stroma into the myometrium. It is a common disease that seriously affects reproductive health of women in childbearing age. Due to the unknown etiology and pathophysiological mechanism, and the lack of unified diagnostic criteria and effective treatment methods, total or subtotal hysterectomy has become a radical treatment for adenomyosis, which will lead to the complete loss of fertility. With the continuous exploration of the treatment to adenomyotic patients who have infertility or fertility intentions, new drugs, surgical methods and treating concepts appears. Adopt individualized conservative therapeutic strategies for patients with different conditions, preserve the uterus as much as possible and protect the patient’s fertility, which will play an important role on the follow-up assisted reproductive treatment and long-term management of adenomyosis.

## 1. Introduction

Adenomyosis is a benign invasion of endometrial glands and stroma into the myometrium, causing myofibrillar connective tissue hypertrophy and hyperplasia, and forming diffuse or localized lesions of the uterus (1). Its main clinical manifestations are progressive dysmenorrhea, uterine enlargement, abnormal uterine bleeding (including increased menstrual volume and prolonged menstrual cycle) and infertility (2). Its occurrence in the infertility population can reach to more than 30%, and it seriously affects the patients’ quality of life. As adenomyosis is more common in women of childbearing age, it has gradually become one of the important clinical problems impairing human fertility, and its incidence rate range from 5 to 70% (3). Severe adenomyosis may be associated with adverse pregnancy and delivery outcomes (4).

### 1.1. Brief introduction of the pathogenesis and diagnosis of adenomyosis

The pathogenesis of adenomyosis is an important basis for treatment, but it has not been fully clarified. There are two main hypotheses about the pathogenesis of adenomyosis (5). One is the endometrial metaplastic hypothesis: adenomyosis lesions are generated directly by the differentiation of ectopic Mullerian tube residual cells, especially pluripotent stem cells with embryonic pluripotent (6). This hypothesis can partly explain the reason why many unmarried young women without pregnancy suffer from adenomyosis.

The other is the endometrial metastatic hypothesis, which believes that the enhancement and disorder of endometrial peristaltic waves, intrauterine infection (including Mycobacterium tuberculosis) or surgical procedures lead to the damage of endometrial-myometrial interface, namely the endometrial myometrium junction zone (JZ); then the glandular cells and stromal cells in the endometrium pass through the endometrial basal layer at the damaged site and invade the myometrium (7, 8). Endometrial stem cells may be recruited and self-repairing mechanism may be initiated in the local lesion. At the same time, the lesion can induce immune imbalance and aseptic inflammation, with increasing proglandin E2 (PGE2), proglandin H2 (PGH2), cyclooxygenase-2 (COX-2) and transforming growth factor β1 (TGF-β1), vascular endothelial growth factor (VEGF) and other factors (5). The increased aromatase activity promotes the synthesis of estradiol, which will up-regulate oxytocin and its receptor, as well as enhance peristalsis and damage of the endometrium (9). In this way, a vicious circle will be created, which eventually caused the cracking of the myometrium, endometrial basal cells invading into the myometrium, and the formation of adenomyosis lesions locally. This hypothesis can explain the clinical manifestations of elevated estrogen, menstrual abdominal pain, and increased menstruation in patients with adenomyosis (5).

In addition, there are other hypotheses, such as the hypothesis of retrograde menstruation, mesothelial metaplasia, etc. (6, 10). So far, no single hypothesis can fully explain all the pathological mechanisms and clinical manifestations of adenomyosis. Recently, investigations on the relationship between colors of human iris and hair and the characteristics of endometriosis may provide new inroads into the molecular aspects of the pathological mechanisms of these complex diseases (11).

At present, there is no consensus on the diagnostic criteria of adenomyosis by imaging methods. The criteria for the diagnosis of adenomyosis based on transvaginal ultrasonography (TVUS) images developed by the Federation International of Gynecology and Obstetrics (FIGO) in 2018 included eight ultrasound features (12, 13): asymmetric thickening of the myometrium, cystic lesions of the myometrium, island hyperechoic signals, fan-shaped shadows, linear or punctate echoes of the endometrium, streaked blood flow signals passing through the lesions, irregular shape and discontinuous JZ. If there are 2 or more of the above signs, the diagnosis can be made in combination with the patient’s clinical manifestations.

MRI features of adenomyosis are of great significance for diagnosis (13, 14). On T2-weighted imaging, localized adenomyosis showed ovoid, irregular or quasi-circular mass in the myometrium, with unclear boundary, low signal similar to the JZ, and scattered dotted or flaky high signal in the lesion, showing cystic expansion or hemorrhage in the lesion. Diffuse adenomyosis showed equal signal on T1-weighted image (T1WI), and dotted high signal in some lesions of T2WI showed that JZ was damaged, showing diffuse thickening. When the thickness of JZ > 12 mm, adenomyosis was highly suspected. If the thickness of JZ is 8–12 mm, combined with high signal spots or irregular boundary, it is proposed to be diagnosed as adenomyosis. Of course, pathological examination through surgery or biopsy is the gold standard for the diagnosis of adenomyosis.

## 2. Individualized oral therapies for adenomyosis

The general therapeutic principles to be followed in clinical practice include reducing and removing lesions, relieving and eliminating pain, improving and promoting fertility, and reducing and avoiding recurrence. Adenomyosis has been regarded as a chronic disease requiring long-term management (15). Most premenopausal women need to receive long-term drug treatment, even after conservative surgery or interventional therapy (16). The individualized treating plan should be made according to the patient’s age, symptoms, ovarian reserve, severity of disease, fertility desire and previous treatments. The main purpose of individualized treatment plan is to solve the problems including chronic pelvic pain, secondary dysmenorrhea, excessive menstruation and infertility.

### 2.1. Non-steroid anti-inflammatory drugs

For patients with the main symptom of pelvic or menstrual pain, non-steroidal anti-inflammatory drugs (NSAIDs) are commonly used if the pain is mild, no fertility requirements or the menopause is approaching. NSAIDs are cyclooxygenase inhibitors, which can reduce the synthesis of prostaglandins in the endometrium and relieve dysmenorrhea (17). However, it cannot prevent the progression of endometriosis and adenomyosis. Adenomyosis relapses easily after NSAIDs withdrawal, and about 18% of patients have no response to NSAIDs (17, 18). Therefore, NSAIDs, such as ibuprofen, is usually used in combination with other treatment methods. It should be noted that long-term use may result to adverse reactions such as peptic ulcer, liver and kidney injury (19).

### 2.2. Compound oral contraceptives

Compound oral contraceptives (COC), such as drospirenone and ethinylestradiol tablets or cyproterone acetate and ethinylestradiol tablets, can reduce the amount of menstruation and relieve dysmenorrhea by inhibiting follicular development and ovulation, reducing estrogen level, inhibiting endometrial proliferation, and promoting endometrial atrophy (19, 20). COC is an important second-line treatment for menorrhagia patients due to adenomyosis, especially for those who need contraception (20). However, because COC has the potential to increase the risk of venous thrombosis and pulmonary embolism, patients over 40 years old or with high-risk factors (such as hypertension, diabetes, thrombosis and smoking history) should be very cautious.

### 2.3. Androgen derivatives

Androgen derivatives including danazol, gestrinone, etc., can lead to endometrial atrophy through double effects (21). On one hand, they inhibit the secretion of follicle stimulating hormone (FSH) and luteinizing hormone (LH), and inhibit ovarian synthesis of steroid hormones (16). On the other hand, they combine with estrogen and progesterone receptors of the endometrium, and finally cause temporary amenorrhea in patients (22). Androgen derivatives have certain effects on dysmenorrhea symptoms and abnormal menstruation (21). However, there is a problem of recurrence after drug withdrawal, and the incidence of androgen like adverse reactions, such as acne and seborrheic dermatitis, weight gain and liver injury.

Mifepristone is a progesterone receptor antagonist with high affinity. It is a derivative of 9-demethyltestosterone, which can be used to terminate pregnancy, soften the cervix, and also cause amenorrhea (23). There have been some studies on the short-term use of low-dose mifepristone in the treatment of adenomyosis with leiomyoma, which has a significant effect on the relief of dysmenorrhea (19). Long term use of mifepristone can result in continuous stimulation of the endometrium by estrogen, so its safety needs further verification.

### 2.4. Synthetic progesterones

Dienogest is a new generation of synthetic progesterone, which has high affinity with progesterone receptor, and has little effect on androgen, glucocorticoid and mineralocorticoid receptor (24). Therefore, it has little effect on organ function and metabolism. Dienogest moderately inhibits the hypothalamus-pituitary-ovary (HPO) axis, and moderately reduces the estrogen level and endometrial proliferation, which prevents the expansion of uterine lesions. The incidence of perimenopausal symptoms after long-term use is low. At the same time, dienogest has anti-inflammatory and anti-angiogenesis effects, as well as promoting the reduction of nerve growth factor expression and nerve fiber density in tissues, which can effectively prevent recurrence and relieve pain (25). Dienogest is mainly suitable for the patients who are in or near the perimenopause with pelvic pain. In addition, the number of invasive NK cells in the endometrial layer increased after dienogest treatment, which may be conducive to the follow-up assisted reproductive treatment of patients with infertility (25, 26). Common adverse reactions include irregular bleeding, breast discomfort, headache, etc., so patients with adenomyosis accompanied by menorrhagia and anemia should be cautious.

The molecular structure of dydrogesterone is similar to that of natural progesterone, but its activity is much higher. Because it has no androgen, estrogen and mineralocorticoid like effects, dydrogesterone does not inhibit ovulation and has no inhibitory effect on normal endometrium. The patient can be pregnant during the medication of dydrogesterone, so it is especially suitable for adenomyosis patients with a recent pregnancy plan (27). Dydrogesterone is rapidly absorbed by oral administration with short half-life, and has obvious effect on inhibiting endometrial proliferation without causing low estrogen symptoms or affecting lipid metabolism and blood system (28). Thus it is safe for long-term use and suitable for patients above 40 years old, or those with high risks of metabolic and thrombotic diseases. Dydrogesterone is also often used in combination with other treatments (29). Due to the widespread use of didroxyprogesterone in other fields of gynecology and reproductive medicine, patients with adenomyosis have higher acceptance and compliance with it (29, 30).

### 2.5. Traditional Chinese medicine

Traditional Chinese medicine has a long history in dealing with various symptoms of adenomyosis. From the perspective of traditional Chinese medicine, the basic pathogenesis of adenomyosis is due to Blood-stasis blocking Chong-ren and Bao-gong, which belongs to internal syndrome (31). The disease location of adenomyosis is in Xia-jiao, so the general treatment principle is to promote blood circulation and remove Blood-stasis (Blood-stasis, Chong-ren, Bao-gong and Xia-jiao are all anatomical and pathological terms of traditional Chinese medicine). Traditional Chinese medicine therapies, such as Guizhi Fuling Capsule, Danhuang Quyu Capsule, Zhitong Huazheng Capsule, Sanjie Zhentong Capsule, Shaofu Zhuyu Decoction Modified, as well as catgut embedding, acupuncture and acupoint application, all have certain effects on relieving the clinical symptoms of adenomyosis (31, 32).

Traditional Chinese medicine is not only beneficial to the treatment of clinical symptoms of adenomyosis, but also provides a completely individualized treatment concept more importantly (33). In fact, traditional Chinese medical treatment is highly individualized for all diseases, including adenomyosis. The types of Chinese herbal medicine and measures used vary from person to person, time to time, and place to place. There is no unified treatment standard, which is instructive for the diagnosis and treatment in modern medicine. There will be a bright future to treat adenomyosis with the concept of combining traditional Chinese and modern medicine.

## 3. Individualized non-oral drugs for adenomyosis

### 3.1. Gonadotropin-releasing hormone agonist

Gonadotropin releasing hormone agonist (GnRH-a) is an category of GnRH analog, whose affinity with GnRH receptor is much higher than that of natural GnRH (34). Long acting GnRH-a binds to a GnRH receptor in the pituitary gland to produce a transient increase in gonadotropin. Its continued binding to the receptor with subsequent movement into the pituitary cell prevents the pituitary gland from responding to endogenous GnRH. In this way, GnRH-a thoroughly inhibits the HPO axis of patients with adenomyosis and induces a sharp decrease in ovarian synthetic steroids, endometrial atrophy, decreased bleeding and even temporary amenorrhea (35). Long-acting GnRH-a can reduce uterine volume of patients with adenomyosis, and dysmenorrhea is also significantly relieved. However, continuous use for several months may lead to perimenopausal symptoms and osteoporosis, and estrogen should be added as appropriate. Because of the high price and obvious recurrence after drug withdrawal, GnRH-a is rarely used alone (36). Although GnRH-a is applicable to a wide range of patients, it is generally used as a pretreatment for other long-term treatments (35).

### 3.2. Levonorgestrel releasing intrauterine system

After levonorgestrel releasing intrauterine system (LNG-IUS) is implanted into the uterus, it can sustain a constant sustained release of 20 μg per day for 5 years. LNG-IUS mainly acts on the endometrium directly, and inhibits the synthesis of estrogen receptor in the endometrium which induces the sensitivity of the endometrium to estradiol and strongly antagonizes the proliferation of the endometrium (37). Therefore, it can be used not only for contraception, but also to treat adenomyosis, reduce the amount of menstruation, shrink the uterine volume, and relieve dysmenorrhea. LNG-IUS is effective for adenomyosis with dysmenorrhea and menorrhagia, especially recommended for the latter (38, 39). LNG rarely enters the peripheral circulation, so there are few systemic side effects except for minor vaginal bleeding and amenorrhea. Thus LNG-IUS can also be used for patients with a history of systemic diseases (such as hypercoagulable state, liver injury) or high risk, but patients must be fully informed of the advantages and disadvantages before use (20). LNG-IUS is applicable to adenomyosis patients who wish to conserve the uterus and fertility. As uterine cavity deformation, poor closure and excessive menstruation are common in adenomyosis patients, LNG-IUS is easy to fall off or move down (40). Accurate positioning under hysteroscopy, fixation to uterine wall, using GnRH-a pretreatment and other measures will help reduce the rate of LNG-IUS shedding and save medical expenses.

Xiao et al. found that LNG-IUS had a good therapeutic effect on patients with small lesions or small uterine volumes, while it had a poor therapeutic effect on patients with large lesions or large uterine volumes, or even no effect (41). This suggests that LNG-IUS is selective for lesion size in the treatment of adenomyosis, that is the phenomenon of ‘lesion threshold’ for LNG-IUS to effectively treat adenomyosis. This may be related to the pharmacological mechanism that LNG directly acts on the endometrium and diffuses to the myometrium. A large number of clinical data shows that the lesion threshold for LNG-IUS to take effect is the lesion range (LR: mean diameter of adenomyoma lesions or thickness of unilateral uterine muscle wall) ≤ 40 mm, and the corresponding threshold of uterine volume is (140.0 ± 35.4) ml. The lesion threshold for marked therapeutic effect is LR < 30 mm, with corresponding uterine volume (117.4 ± 34.1) ml, so that LNG-IUS had the most obvious effect in relieving symptoms and reversing the condition. When LR is 30 ~ 40 mm, LNG-IUS can reduce menstruation and dysmenorrhea, but the effect on reducing the uterine volume is not satisfactory. When LR > 40 mm, LNG-IUS alone has nearly no effect on reducing symptoms (41, 42). Lee et al. also showed that the effect of LNG-IUS on adenomyosis was poor if the uterine volume was >150 ml (43). We hope that there will be more *in vivo* and *in vitro* researches on the LR of LNG-IUS in the future, which will provide a research basis for the wider clinical application of LNG-IUS.

### 3.3. Other drugs

The main component of subdermal implant contraceptives is also synthetic potent progestogen which can achieve a theoretical effect similar to LNG, but its clinical application is limited (44).

## 4. High intensity focused ultrasound

High intensity focused ultrasound (HIFU) is a kind of physical therapy, which has the advantages of no operation, no bleeding, no ionizing radiation and repeatability (38). It is a more accurate treatment for localized adenomyosis with good effect, and preserves the integrity of the uterus. It is suitable for patients who have fertility planning and desire to conserve the uterus. The principle of HIFU ablation is to focus the ultrasound on the target tissue through the instrument. The thermal effect, cavitation effect and other physical effects of the ultrasound instantly raise the temperature of the target tissue to 65°C ~ 100°C, and cause tissue protein denaturation, coagulative necrosis. The tissue finally is dissolved, absorbed, calcified or fibrotic (41). With the continuous improvement of technology, the popularity and indications of HIFU treatment are gradually expanding, but it is easy to recur. In most cases it needs to be used in combination with other methods (45).

## 5. Conservative surgical management for adenomyosis

The uterus is not only a reproductive organ, but also plays a role in maintaining the normal anatomical structure of the pelvic floor as well as the blood supply of pelvic organs, especially ovary. Radical surgery, including total hysterectomy or subtotal hysterectomy, is the most thorough and effective method to treat adenomyosis, but it will deprive the fertility and have adverse effects on patients’ pelvic floor function, ovarian function, sexual life quality and self-cognition (46). Therefore, more and more attention has been paid to the conservative surgery.

Uterine artery embolization (UAE) and laparoscopic uterine artery occlusion on patients with adenomyosis both aim to block the blood supply of the lesion, but may lead to obstruction of ovarian blood supply or endometrial necrosis, resulting to secondary infertility (47). Transcervical endometrial resection (TCER) can lessen the symptoms of adenomyosis but cannot interfere with its progress. TCER removes the material basis of embryo implantation, so it cannot be used for patients with fertility requirements (48).

Resection of uterine lesion (RUL) is a very complex and challenging operation, and to achieve a balance between removing adenomyosis and retaining normal myometrium and the integrity of the uterus (49). RUL is often applied to adenomyosis patients with fertility requirements or young age. Therefore, there is no completely rigid surgical procedure, and flexible operations should be carried out according to factors such as the shape of the uterus, the size and location of the lesions. Common operational methods include wedge resection, asymmetric resection, and partial resection of muscle wall lesions (50). The repair of uterine myometrium and the reconstruction of uterine body are crucial for the uterus to withstand pregnancy, including H-shaped and, U-shaped suture, muscle overlapping-flap suture and triple-flap suture proposed by Osada, etc. (51, 52). In recent years, Xiao et al. have created the major uterine wall resection and reconstruction of the uterus (MURU) on the basis of RUL, which provides a new surgical method for patients with severe adenomyosis to preserve the uterus (42). MURU destroys the structure of the uterus, and the uterine scar is large with the high risk of uterine rupture during pregnancy. Therefore we are modifying the procedures to meet the fertility requirements of patients after operation. Of course, multi-center clinical research and more verification are needed before large-scale promotion of MURU. However, the recurrence, low natural pregnancy rate and the risk of uterine rupture during pregnancy are the main problems after conservative surgery (53). It is very important to treat as early as possible and develop an individual combined therapy to preserve the uterus according to the degree of adenomyosis.

## 6. Individualized combined therapy

Different therapies for adenomyosis have their own advantages and disadvantages, which causes great confusions for doctors and patients and probably leads to insufficient or excessive treatments (Table 1). Xiao et al. proposed a personalized therapeutic strategy based on the concept of grading, which provides a new option for patients with adenomyosis who wish to preserve fertility (41, 42). It divides adenomyosis into three grades according to the LR: mild (LR ≤ 30 mm), medium (LR between 30-40 mm) and severe (LR ≥ 40 mm). The corresponding processing methods for each grade are as follows:

**Table 1:** The characteristics and application of individualized fertility preserving treatments for adenomyosis.

Classification of treatments		Typical drugs	The characteristics and applications	Special notes
Individualized oral therapies	NSAIDs	Ibuprofen	Reliese the pelvic or menstrual pain; Used in combination with other treatment methods	18% of patients no response; Long-term use may result to peptic ulcer, liver and kidney injury, etc.
	COC	Trospirone ethinylestradiol tablets; Ethinylestradiol cycloproterone tablets	Reduce menstruation; Relieve dysmenorrhea; Inhibit endometrial proliferation; Fit for contraception	Increase the risk of thrombosis and embolism; Use with cautions for patients over 40-y or with high-risk factors
	Androgen derivatives	Danazol; Gestrinone; Mifepristone; etc	Inhibit the secretion of gonadotropins and sex hormones and induce temporary amenorrhea	Recurrence after drug withdrawal; Androgen like adverse reactions; Security risk for long-term use
	Synthetic progesterones	Dienogest; Dydrogesterone	Inhibit HPO axis, Reduces the estrogen level and endometrial proliferation, Relieve pain, Fit for perimenopause patients	Adverse reactions include irregular bleeding, breast discomfort, headache, etc.,
	Traditional Chinese medicine	Guizhi Fuling Capsule; Danhuang Quyu Capsule; Zhitong Huazheng Capsule; Sanjie Zhentong Capsule; Shaofu Zhuyu Decoction Modified	Promote blood circulation; Remove Blood-stasis; Relieving clinical symptoms	Provides completely individualized treatments
Individualized non-oral therapies	GnRH-a	Leuprorelin Acetate Microspheres For Injection	Thoroughly inhibit the HPO axis; Reduce ovarian synthetic steroids and uterine volume; Relieve pain	Long-term use leads to perimenopausal symptoms; Obvious recurrence after withdrawal; Used as pretreatment
	LNG-IUS	LNG-IUS	Act on the endometrium directly; Antagonize endometrial proliferation; Few systemic side effects	Easy to fall off or move down; Minor vaginal bleeding and amenorrhea
	Subdermal implant contraceptives	Levonorgestrel Silastic Implants	Similar to LNG-IUS	Limited clinical application
HIFU		–	The thermal and cavitation effect cause tissue protein denaturation and finally dissolution or fibrosis	A physical therapy with no operation; Fit for localized adenomyosis; Easy to recur
Conservative surgery	Uterine artery embolization; Laparoscopic uterine artery occlusion	–	Block the blood supply of the lesion in uterus	May lead to obstruction of ovarian blood supply or endometrial necrosis
	Transcervical endometrial resection	–	Lessen the symptoms of dysmenorrhea	Unable to prevent disease progression and damage fertility
	Resection of uterine lesion	–	Flexible operations according to the shape of the uterus, the size and location of the lesions; Include H-shaped, U-shaped, muscle overlapping-flap, triple-flap and Xiao’s MURU suture	Recurrence, low natural pregnancy rate and risk of uterine rupture during pregnancy

(1) For patients of mild grade, the long-term management by LNG-US is the main method. If there are still symptoms such as dysmenorrhea after that, NSAIDs, progesterone and COC should be given. Remove LNG-US before receiving assisted reproductive therapy.
(2) For patients of moderate grade, GnRH-a can be used for 3–6 months firstly. If LR reduces to ≤30 mm, the patients should be treated according to the principle of mild grade. If LR does not reduce obviously, HIFU can be used for to reduce LR as combined treatment.
(3) For patients of severe grade, GnRH-a should also be used for 3–6 months firstly. RUL or modified MURU should be performed after GnRH-a, and LNG-US is placed into the uterine cavity during the surgery. Remove LNG-US before receiving assisted reproductive therapy.

This grading of adenomyosis is mainly based on the LR in imaging, which lacks evaluation of clinical symptoms of patients, and needs further development to be widely used in practice. Anyway, this grading strategy is a great promotion to the treatment of adenomyosis.

## 7. Conclusion

Adenomyosis is a disease that tends to occur in childbearing aged women. The treatments are diverse but lack specificity. The basic treating principles for the adenomyosis patients with fertility requirements should include protecting physical and mental health, preserving reproductive organs and functions. It is crucial to allow patients the full advantages of each method and choose an individualized strategy according to the grading and needs of each patient. Follow up should be insisted, and combined or continuous therapy should be used to relieve symptoms and prevent disease progression if necessary.

## Author contributions

LH and YL determined the topic and write the manuscript. KL and JJ retrieved literatures. CZ made the table. CZ and YW analyzed data and revised the manuscript. All authors contributed to the article and approved the submitted version.

## Funding

This work was supported by the Scientific Research Fund of Binzhou Medical University (BY2021KYQD34); Scientific Research Initiation Fund of Affiliated Hospital of Binzhou Medical University Hospital (2021-03); Science and Technology Plan Project of Honghuagang District in Zunyi City (Zun Hong Ke He She[2020] No. 07); Science and Technology Plan Project of Zunyi City (Zun Yi Ke He HZ[2020] No. 271).

## Conflict of interest

The authors declare that the research was conducted in the absence of any commercial or financial relationships that could be construed as a potential conflict of interest.

## Publisher’s note

All claims expressed in this article are solely those of the authors and do not necessarily represent those of their affiliated organizations, or those of the publisher, the editors and the reviewers. Any product that may be evaluated in this article, or claim that may be made by its manufacturer, is not guaranteed or endorsed by the publisher.
